# A new nomogram to predict oncological outcome in laryngeal and hypopharyngeal carcinoma patients after laryngopharyngectomy

**DOI:** 10.1007/s00405-022-07668-1

**Published:** 2022-10-01

**Authors:** Stefan Grasl, Florian Frommlet, Muhammad Faisal, Blazen Marijic, Elisabeth Schmid, Gregor Heiduschka, Markus Brunner, Matthaeus C. Grasl, Boban M. Erovic, Stefan Janik

**Affiliations:** 1 Department of Otorhinolaryngology, Head and Neck Surgery, Medical University of Vienna, Waehringer Guertel 18-20, Vienna, Austria; 2 Center for Medical Statistics, Informatics and Intelligent Systems, Section for Medical Statistics, Medical University Vienna, Vienna, Austria; 3 Shaukat Khanum Memorial Cancer Hospital, Lahore, Pakistan; 4 Department of Otorhinolaryngology, Head and Neck Surgery, Clinical Hospital Center Rijeka, Rijeka, Croatia; 5 Institute of Head and Neck Diseases, Evangelical Hospital, Vienna, Austria

**Keywords:** Laryngectomy, Laryngopharyngectomy, Oncological outcome, Nomogram, Cancer

## Abstract

**Background:**

To create nomograms for better prediction of the oncological outcome in advanced laryngeal (LxCAs) or hypopharyngeal (HpxCAs) cancer after laryngopharyngectomy.

**Materials:**

239 patients who underwent total laryngectomy or laryngopharyngectomy due to LxCA (52.7%) or HpxCA (47.3%) were included in this study. Based on clinical risk factors (tumor site, lymph node involvement, salvage setting), we created nomograms for prediction of disease-specific survival (DSS) and disease-free survival (DFS).

**Results:**

HpxCAs showed a higher rate of lymph node involvement (*p* < 0.001), a 2.47-fold higher risk of a 2nd head and neck cancer (*p* = 0.009) and significantly worse loco-regional control rates (*p* = 0.003) compared to LxCAs. Positive neck nodes and salvage procedures were associated with significantly worse outcome. Nomograms demonstrated that hypopharyngeal tumors with positive neck nodes in salvage situations had the worst oncological outcome with a 5-year DSS of 15–20%.

**Conclusions:**

The oncological outcome is worse in hypopharyngeal carcinomas and could be easily quantified by our nomograms that are based on tumor site, lymph node involvement and salvage situation.

## Introduction

The treatment of advanced laryngeal (LxCA) and hypopharyngeal carcinomas (HpxCA) is a balance between oncological safety and functional preservation to enable an acceptable quality of life [1–3]. Since the early 1990s, two different approaches have been established for both tumor entities defined as non-organ-preserving and organ-preserving protocols. The latter comprises primary chemoradiotherapy (CRT), while non-organ-preserving approaches are based on tumor removal through total laryngectomy (TL) or laryngopharyngectomy mostly followed by radiotherapy (RT) [4–6].

However, the oncological outcome of advanced stage LxCA and HpxCA is still poor with a 5-year overall survival of around 50–65% [7]. Locoregional and distant recurrence which range from 25% to 50% are the major prognostic determinants and main predictors of mortality [8, 9]. Despite their close anatomical proximity and similar treatment approaches, outcomes seem to be remarkably worse in hypopharyngeal carcinomas [8–10]. A higher ratio of lymph node metastasis is considered as main predictor for poor outcome of HpxCAs followed by advanced-stage disease, incomplete tumor resection and extracapsular extension [11–13].

Notably, outcome analyses comparing both tumor entities undergoing total laryngectomy in salvage and non-salvage situations are lacking [14]. Therefore, the main objectives of the study were to evaluate the oncological outcomes in advanced hypopharyngeal or laryngeal cancer patients who underwent laryngectomy, identify potential risk factors contributing to poor outcome and to create a nomogram based on those variables that might be helpful for more precise prediction of future patients’ oncological outcome.

## Materials and methods

### Study cohort

Between 1993 and 2020, 239 patients underwent total laryngectomy (TL; *n* = 76; 31.8%) or total laryngopharyngectomy (TLTP; *n* = 163; 68.2%) due to histologically verified squamous cell carcinomas (SCCs) of the larynx (*n* = 126; 52.7%) or hypopharynx (*n* = 113; 47.3%). All patients were treated at the Department of Otorhinolaryngology, Head and Neck Surgery of the Medical University of Vienna, Austria with a mean follow-up time of 49.1 ± 59.6 months.

After discussion in the multidisciplinary tumor board, TL or TLTP was either performed as a primary or salvage surgery. Infiltration of the vertebral fascia or the common/internal carotid artery represented contraindications for a surgical procedure. Those patients who opted for primary radiochemotherapy were treated with concomitant platin-based chemotherapy, which is the current standard of care. Concomitant Cetuximab was applied in elderly patients (≥ 75 years) and those with contraindications for platin-based chemotherapy.

### Clinical data

Clinical and sociodemographic characteristics for each patient were obtained from medical hospital records, surgical and pathological reports, as well as imaging findings. We were particularly interested in the extent of surgery (TL vs. TLTP), tumor extension (T-classification, N-classification, AJCC tumor stage), occurrence of complications and previous treatment regimens. Reported TNM staging represents the final pathological report of the primary or salvage surgery. Per definition, resection margins ≤ 5 mm were considered as positive [15].

### Oncological outcomes

We used the disease-specific survival (DSS), the disease-free survival (DFS) and incidence of recurrences or second malignancies as oncological outcome parameters. DSS was calculated from date of surgery to date of death from HpxCA or LxCa. Unrelated deaths, unknown reasons for death or deaths due to another malignant disease represented censored events. Otherwise, DFS was calculated solely in patients who were assumed to be “free of cancer” ranging from date of surgery to date of recurrence. The latter were further differentiated into local, regional and/or distant failures. As there is no widely accepted definition for whether a tumor represents a secondary primary HNSCC or locally recurrent cancer, we considered cancers occurring more than 60 months after initial therapy as 2nd primary HNSCC [16].

### Statistical methods

Statistical analyses were performed using SPSS version 27.0 software (IBM SPSS Inc., Armonk, NY, USA) and R version 3.6 [R Core Team (2019). R: a language and environment for statistical computing. R Foundation for Statistical Computing, Vienna, Austria]. Unless otherwise specified, data are reported as mean ± standard deviation (SD). Descriptive statistics were used for analysis of demographic and clinical data. Chi-square test was used to investigate the association between nominal variables. Unpaired Student’s *t* test was used to compare means of two independent groups with normal (Gaussian) distributions. Kaplan–Meier analysis and Log-rank test were assessed for univariate outcome analysis. Uni- and multivariate cox regression analyses were used to evaluate the prognostic impact of different clinical variables on DSS and DFS. Hazard Ratios (HRs) and corresponding 95% confidence intervals (CIs) are indicated. All tests were performed two-sided and *p* values below 0.05 were considered as statistically significant. To create nomograms for DSS and DFS, we performed variable selection among all potential predictor variables using all subset selection based on the Akaike information criterion (AIC). The final best Cox regression models were visualized with two nomograms including 6-, 12-, 24- and 60-month survival using the R package “rms” [17]. For an internal validation method, we provided numeric (Harrell’s c-index 0.642 for DSS and 0.621 for DFS) and graphical (calibration curve according to Austin et al. [18]) information on the discriminative and predictive accuracy of the nomograms presented herein. For the survival analyses and nomograms, the absence (N0) and extent (N1–3) of regional lymph node involvement according to the TNM classification was summarized as N− and N + .

## Results

### Patient cohort

In total, 239 patients were included comprising 25 females (10.5%) and 214 males (89.5%) with a mean patient age of 59.1 ± 9.3 years. Among them, 126 patients (52.7%) had LxCAs and 113 (47.3%) suffered from HpxCAs. The majority of SCCs showed moderate differentiation (68.2%). BMI, age and gender distribution were significantly different among laryngeal and hypopharyngeal tumors (*p* < 0.001; *p* = 0.002; *p* = 0.028). The amount of nicotine (67.3% vs. 54.0%) and alcohol abuse (55.8% vs. 38.9%) was significantly higher in HpxCAs than in LxCAs (*p* = 0.036; *p* = 0.009; Table 1).

**Table 1:** Patient cohort

Variables	Overall	Larynx	Hypopharynx	*p* value
	*n (%)*	*n (%)*	*n (%)*	
Sex	239 (100)	126 (52.7)	113 (47.3)	
Female	25 (10.5)	8 (6.3)	17 (15)	
Male	214 (89.5)	118 (93.7)	96 (85)	**0.028**^**a**^
Age (mean ± SD)	59.1 ± 9.3	60.9 ± 9	57.1 ± 9.3	**0.002**^**b**^
Body-mass-index (BMI)				
Before Surgery	23.7 ± 4.5	24.8 ± 4.8	22.5 ± 3.7	**< 0.001**^b^
Nicotine abuse				
No	95 (39.7)	58 (46)	37 (32.7)	
Yes	144 (60.3)	68 (54)	76 (67.3)	**0.036**^**a**^
Alcohol abuse				
No	102 (53.1)	77 (61.1)	50 (44.2)	
Yes	112 (46.9)	49 (38.9)	63 (55.8)	**0.009**^**a**^
T-classification*				
T1	9 (3.8)	1 (0.8)	8 (7.1)	
T2	33 (13.8)	15 (11.9)	18 (15.9)	
T3	87 (36.4)	49 (38.9)	23 (33.6)	
T4a	110 (46)	61 (48.4)	46 (43.4)	0.052^a^
N-classification*				
N0	98 (41)	69 (54.8)	29 (25.7)	
N1	21 (8.8)	8 (6.3)	13 (11.5)	
N2	87 (36.4)	32 (25.2)	55 (48.7)	
N3	9 (3.8)	3 (2.4)	6 (5.3)	**< 0.001**^**a**^
Nx	24 (10.0)	14 (11.1)	10 (8.8)	
Grading				
G1	19 (7.9)	14 (11.1)	5 (4.4)	
G2	163 (68.2)	87 (69)	76 (67.3)	
G3	57 (23.8)	25 (19.8)	32 (28.3)	0.075^a^
Margins				
R0	210 (87.9)	115 (91.3)	95 (84.1)	
R1	24 (10)	8 (6.3)	16 (14.2)	
R2	5 (2.1)	3 (2.4)	2 (1.8)	0.130^a^
Salvage situation				
Yes	83 (34.7)	42 (33.3)	41 (36.3)	
No	156 (65.3)	84 (66.7)	72 (63.7)	0.632^a^
Surgery				
TL	76 (31.8)	74 (58.7)	2 (1.8)	
TLTP	163 (68.2)	52 (41.2)	111 (98.2)	**< 0.001**^**a**^
Pharynxreconstruction				
Primary closure	160 (66.9)	112 (88.9)	48 (42.5)	
Flap reconstruction	79 (33.1)	14 (11.1)	65 (57.5)	**< 0.001**^**a**^
Neck dissection				
No	45 (18.8)	25 (19.8)	20 (17.7)	
Yes	194 (81.2)	101 (80.2)	93 (82.3)	0.672^a^
Preoperative tracheostomy				
No	171 (71.5)	83 (65.9)	88 (77.9)	
Yes	68 (28.5)	43 (34.1)	25 (22.1)	**0.040**^**a**^
PORT				
No	96 (40.2)	53 (42.1)	43 (38.1)	
Yes	143 (59.8)	73 (57.9)	70 (61.9)	0.528^a^

Bold indicates *p* < 0.05

*SD* standard deviation, *a* Chi-square test, *b* Unpaired students *T* test, *T* T-classification of primary tumor according to TNM classification, *N* N-classification of regional lymph node metastasis according to TNM classification, *PORT* postoperative radiotherapy, *TL* total laryngectomy, *TLTP* total laryngopharyngectomy

### Oncological and histopathological characteristics

We had 9 (3.8%) T1, 33 (13.8%) T2, 87 (36.4%) T3 and 110 (46%) T4a tumors. Thereof 98 patients (45.6%) presented with a N0 neck followed by N2, N1 and N3 necks in 87 (40.5%), 21 (9.8%), and 9 (4.2%) cases, respectively. While the T-classification was short of being significantly different among HpxCA and LxCA (*p* = 0.052), lymph node metastases (N1–N3) was significantly more common in HpxCAs (74.3% vs. 45.2%; *p* < 0.001). In particular, only 19.6% of primary HpxCAs presented with N0 necks compared to 80% in primary LxCAs (Table 2).

**Table 2:** Analysis of lymph node involvement of primary carcinomas according to tumor localization and tumor classification

	N0			N1			N2			N3		
	*LX*	*HPX*	*p*	*LX*	*HPX*	*p*	*LX*	*HPX*	*p*	*LX*	*HPX*	*p*
T1–2	2 (*4.4*)	3 (*27.3*)		1 (2*0*)	3 (33.3)		4 (13.8)	8 (17.4)		0 (0)	2 (*33.3*)	
T3–4	43 (*95.9*)	8 (*72.7*)	**0.017**^a^	4 (*80*)	6 (*66.7*)	0.597^a^	25 (*86.2*)	38 (82.6)	0.679^a^	3 (100)	4 (*66.7*)	0.257^a^
Total	45 (80.4)	11 (19.6)	< **0.001**^a^	5 (35.7)	9 (64.3)	< **0.001**^a^	29 (38.7)	46 (61.3)	< **0.001**^a^	3 (33.3)	6 (66.7)	< **0.001**^a^

Bold indicates *p* < 0.05

*T* T-classification of primary tumor according to TNM classification, *N* N-classification of regional lymph node metastasis according to TNM classification, *LX* larynx, *HPX* hypopharynx, *p* *p* value, *a* Chi-square test

### Extent of surgery

TLs were done in 58.7% of LxCAs, while TLTPs resulting in circumferential defects were performed in 52.2% of HpxCAs (*p* < 0.001). Subsequently, pharyngeal reconstruction with free flaps was significantly more frequent in hypopharyngeal tumors (57.5% vs. 11.1%; *p* < 0.001). Altogether, free resection margins were achieved in 87.9% of cases. Preoperative tracheostomy was more commonly present in LxCAs (34.1% vs. 22.1%; *p* = 0.040) and in cases resulting in incomplete tumor resections (*p* = 0.018). 24.8% of HpxCAs had undergone previous chemo-/immunotherapy, which was significantly higher compared to 11.9% in LxCAs (*p* < 0.001). However, salvage rates, indicating former radiotherapy, were similar between HpxCAs and LxCAs (36.3% vs. 33.3%; *p* = 0.632).

### Oncological outcome

Since there were significant differences regarding extent of surgery, previous treatment regimens and TNM-classification, we were further interested in whether the oncological outcome differs among patient cohorts.

Recurrences occurred in 52.2% of hypopharyngeal tumors compared to 36.5% in laryngeal tumors (*p* = 0.015). The risk of loco-regional failures was 1.7-times higher in HpxCAs (40.7% vs. 23.0%; *p* = 0.003) accompanied by a trend toward higher risk for distant failures (*p* = 0.058) as well. Hypopharyngeal cancer patients also carried a 2.4-fold higher risk for the development of a 2nd head and neck cancer (19.5% vs. 7.9%; *p* = 0.009); (Table 3).

**Table 3:** Oncological Outcome, Recurrences, 2^nd^ Carcinoma and Survival

Variables	Overall	Larynx	Hypopharynx	*p* value
	*n (%)*	*n (%)*	*n (%)*	
Recurrence				
No	134 (56.1)	80 (63.5)	54 (47.8)	
Yes	105 (43.9)	50 (36.5)	59 (52.2)	**0.015**^**a**^
Distant failure				
No	194 (81.2)	108 (85.7)	86 (76.1)	
Yes	45 (18.8)	18 (14.3)	27 (23.9)	0.058^a^
Locoregional failure				
No	164 (68.6)	97 (77)	67 (59.3)	
Yes	75 (31.4)	29 (23)	46 (40.7)	**0.003**^**a**^
2nd cancer				
No	181 (75.7)	101 (80.2)	80 (70.8)	
Yes	58 (24.3)	25 (19.8)	33 (29.2)	0.092^a^
2nd Cancer NSCLC				
No	224 (93.7)	117 (92.9)	107 (94.7)	
Yes	15 (6.3)	9 (7.1)	6 (5.3)	0.560^a^
2nd Cancer Head& Neck				
No	207 (86.6)	116 (32.1)	91 (80.5)	
Yes	32 (13.4)	10 (7.9)	22 (19.5)	**0.009**^**a**^
Survival outcome				
Alive	69 (28.9)	41 (32.5)	28 (24.8)	
Dead	170 (71.1)	85 (67.5)	85 (75.2)	0.186^a^
Cancer	109 (45.6)	47 (37.3)	62 (54.9)	
Other cancer	21 (8.8)	12 (9.5)	9 (8)	
Other reason	40 (16.7)	26 (20.6)	14 (12.4)	**0.049**^**a**^

Bold indicates *p* < 0.05

*a* Chi-square test, *NSCLC* non-small-cell lung carcinoma

### Survival analyses and prognostic factors

The more aggressive oncological behavior of HpxCAs was also reflected in survival analyses, showing that the DSS and DFS were significantly worse in patients with hypopharyngeal tumors (*p* = 0.013; *p* = 0.013). Positive neck nodes (*p* = 0.001; *p* = 0.004) and salvage procedures (*p* = 0.003; *p* = 0.022) were further associated with significantly worse outcome (Table 4).

**Table 4:** Survival analysis

Variables	Disease-specific survival				Disease-free survival			
	1y	3y	5y	*p*^a^	1y	3y	5y	*p*^a^
Location								
Larynx	86.6	64.5	56.2		80.3	61.7	58.6	
Hypopharynx	79.0	48.1	40.1	**0.013**	64.7	48.4	42.7	**0.013**
Tumor staging								
I–III	86.9	68.2	58.8		80.9	64.7	57.3	
IV	81.3	51.8	44.1	**0.034**	69.2	51.3	48.1	0.101
pT classification								
pT1–2	77.3	51.6	44.2		70.8	48.5	44.7	
pT3–4	84.2	57.6	49.2	0.518	73.2	56.6	52.0	0.450
pN classification								
pN−	90.1	76.0	63.7		83.7	70.1	63.7	
pN+	79.8	46.7	41.5	0.001	69.1	48.4	45.0	0.004
Grading								
G1–G2	86.2	56.1	47.1		73.6	53.2	48.3	
G3	72.7	58.2	52.5	0.885	70.0	62.8	59.7	0.439
Margin status								
Negative (R0)	85.1	59.4	50.0		75.4	56.3	51.4	
Positive (R1)	77.8	44.9	44.9	0.352	50.4	45.3	45.4	0.201
Salvage								
No	87.9	62.6	53.2		77.9	60.9	54.5	
Yes	73.5	45.1	39.3	**0.003**	62.5	43.9	43.9	0.022

Bold indicates *p* < 0.05

*p* *p* value, *y* years, *a* Log-rank test, *T* T-classification of primary tumor according to TNM classification, *N* N-classification of regional lymph node metastasis according to TNM classification summarized as pN− (N0) and pN + (N1–3)

**Fig. 1:**
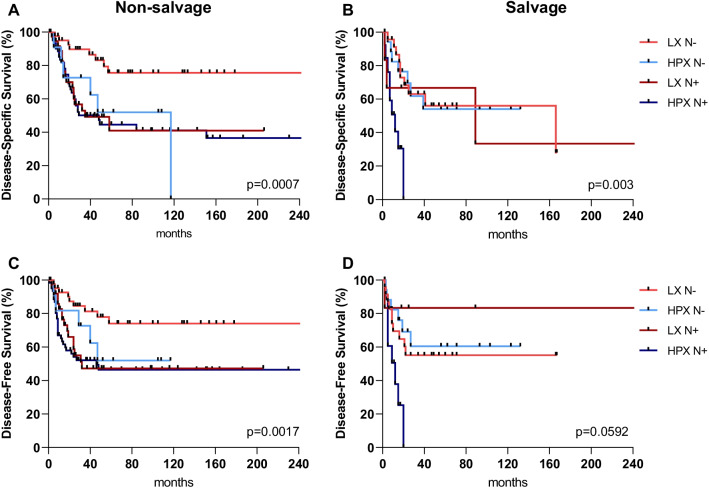
Survival curves. Kaplan Meier survival curves showing disease-specific survival (**A** + **B**) and disease-free survival (**C** + **D**) according tumor location and lymph node involvement and salvage (**B** + **D**) or non-salvage situation (**A** + **C**)

We further differentiated between salvage and non-salvage procedures and whether laryngeal and hypopharyngeal tumors presented with (N+) or without (N−) lymph node metastasis. LxCA patients without neck metastasis (N−) who underwent primary laryngectomy showed the best oncological outcome with a 5-year DFS and DSS of 74.0% and 75.9%. In contrast, the worst oncological outcome with a 5-year DFS and DSS of 0%, was seen in lymph node positive (N+) hypopharyngeal tumors in salvage situations (Fig. 1).

Both, positive neck nodes and salvage procedures further turned out to represent independent prognosticators for poor DSS (HR 2.22; *p* = 0.006; HR 2.08; *p* = 0.005) and DFS (HR 2.08; *p* = 0.005; HR 1.72; *p* = 0.006) as well (Table 5).

**Table 5:** Univariable and multivariable cox-regression analyses

Clinical characteristics	Univariable			Multivariable		
	*p* value	HR	95% CI	*p* value	HR	95% CI
Disease-specific survival						
Location (Larynx vs. Hypopharynx)	**0.014**	0.62	0.43–0.91	0.199	0.75	0.44–1.05
Tumor staging (I–III vs. IV)	**0.037**	0.62	0.39–0.97	0.406	0.78	0.43–1.41
T-classification (T3–4 vs. T1–2)	0.521	0.85	0.53–1.39			
N-classification (N− vs. N +)	**0.001**	0.48	0.31–0.75	**0.006**	0.45	0.26–0.80
Grading (G1–G2 vs. G3)	0.886	1.03	0.66–1.61			
Salvage (No vs. Yes)	**0.004**	1.77	1.21–2.60	**0.005**	0.48	0.29–0.80
Margins (R0 vs. R1)	0.357	0.75	0.41–1.38			
Age (< 60 vs. > 60 years)	0.884	0.97	0.67–1.42			
Gender	0.553	0.86	0.51–1.43			
Pre OP TT (No vs. Yes)	**0.041**	0.66	0.44–0.98	0.274	0.77	0.48–1.23
Complications (No vs. Yes)	**0.007**	0.59	0.40–0.86	0.160	0.73	0.46–1.14
Disease-free survival						
Location (Larynx vs. Hypopharynx)	**0.015**	0.61	0.41–0.91	0.242	0.76	0.48–1.20
Tumor staging (I–III vs. IV)	0.108	0.69	0.44–1.09			
T-classification (T3–4 vs. T1–2)	0.457	1.21	0.73–1.99			
N-classification (N− vs. N +)	**0.005**	0.53	0.34–0.83	**0.005**	0.48	0.29–0.80
Grading (G1–G2 vs. G3)	0.446	0.83	0.51–1.35			
Salvage (No vs. Yes)	**0.025**	0.63	0.42–0.95	**0.036**	0.58	0.35–0.97
Margins (R0 vs. R1)	0.210	0.68	0.37–1.24			
Age (< 60 vs. > 60 years)	0.795	1.05	0.71–1.56			
Gender	0.626	0.84	0.43–1.67			
Pre OP TT (No vs. Yes)	0.296	0.79	0.51–1.22			
Complications (No vs. Yes)	**0.049**	0.66	0.44–1.00	0.388	0.81	0.51–1.30

Bold indicates *p* < 0.05

*HR* hazard ration, *95% CI* 95% confidence interval, *T* T-stage or extent of primary tumor according to TNM classification, *N* N-classification of regional lymph node metastasis according to TNM classification summarized as N− (N0) and N+ (N1–3), *TT* tracheostomy

The overall DSS and DFS did not significantly change over the past three decades (*p* = 0.591; *p* = 0.642). Separate analysis of LxCAs and HpxCAs also revealed no statistically significant change during the observation period (*p* = 0.135; *p* = 0.418 and *p* = 0.117; *p* = 0.250).

### Nomogram

**Fig. 2:**
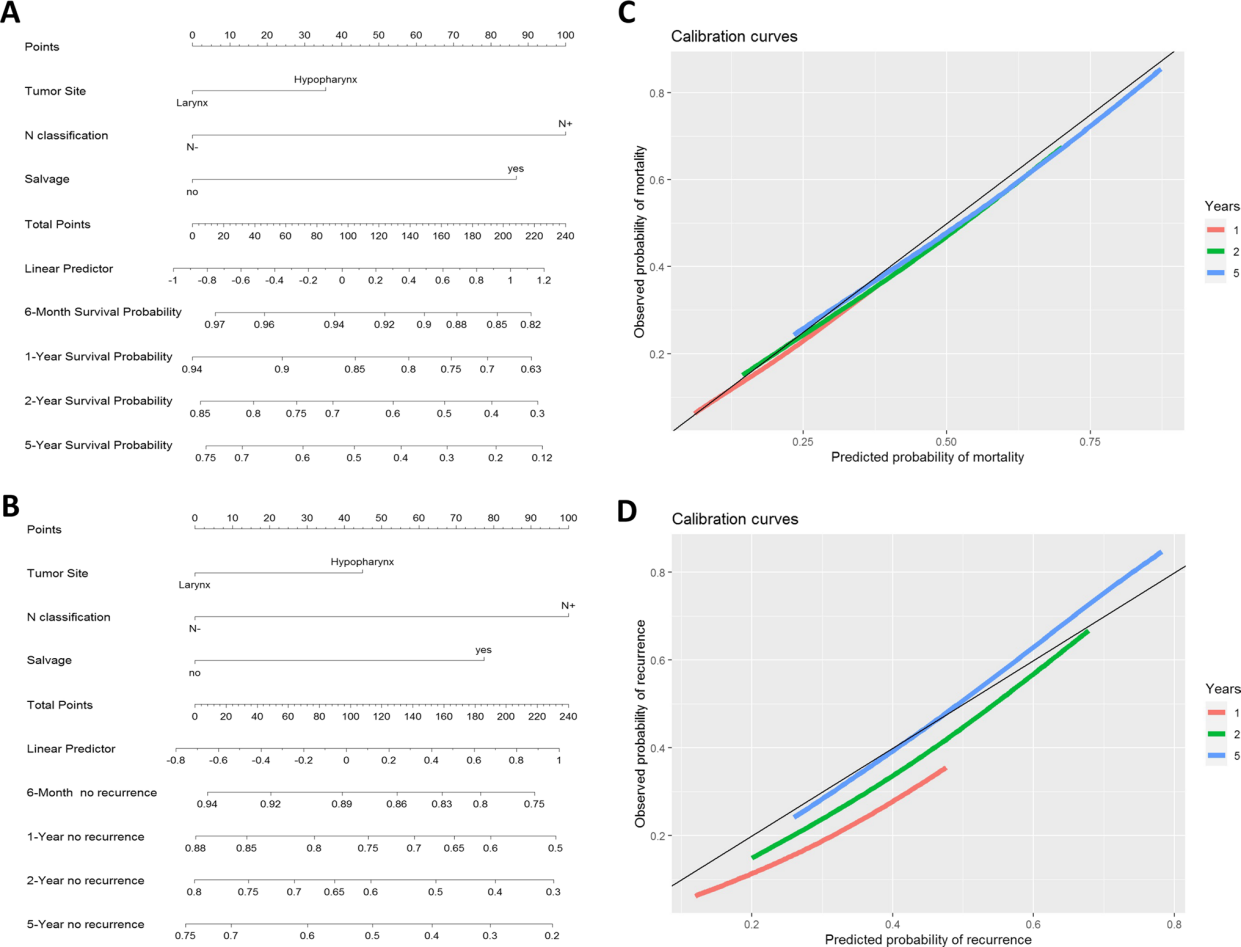
Nomograms. Nomograms to predict 6-, 12-, 24- and 60-month disease-specific survival (**A**) and disease-free survival (**B**) with corresponding calibration curves (**C**, **D**) in advanced staged laryngeal and hypopharyngeal cancer, respectively. The calibration curves were calculated based on the following calculations of van Klaren et al. [18]. Tumor site, lymph node involvement (N classification), and salvage situation were significant factors in our model

**Fig. 3:**
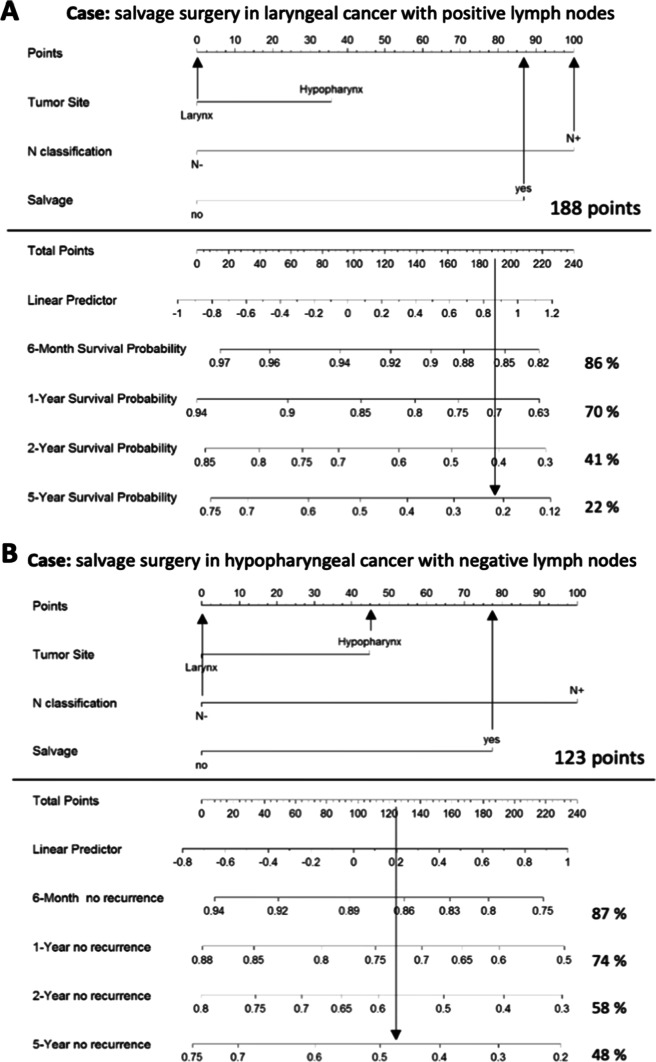
Specific cases. Specific cases illustrate how to use these nomograms to obtain the respective survival probabilities. Part A shows our nomogram for disease-specific survival (DSS) and part B the nomogram for disease-free survival (DFS)

Finally, we created nomograms for better prediction of DSS and DFS for laryngeal and hypopharyngeal cancer patients undergoing ablative surgery (Fig. 2). Anatomic subsite (larynx vs. hypopharynx), N-classification (N− vs. N+) and salvage situation (Yes vs. No) were identified as predictors. Altogether, our nomograms indicate that patients with hypopharyngeal tumors with lymph node involvement (N+) who undergo salvage laryngectomy have the worst 5-year DFS (occurrence of recurrence) and DSS of 15–20% and 10–15%, respectively. Specific cases illustrate how to use these nomograms to obtain the respective survival probabilities (Fig. 3).

## Discussion

Laryngectomy and laryngopharyngectomy are principally performed in advanced laryngeal and hypopharyngeal carcinomas with curative intent. Despite anatomic proximity and identical surgical procedures, outcome of both tumor entities differs tremendously. Nonetheless, corresponding reports comparing oncological endpoints of these tumor entities are lacking [14, 19, 20]. Recently, we demonstrated that laryngopharyngectomies carry a high risk of complications that was directly linked to the extent of ablative surgery accompanied by gradual decrease of functional outcome [3, 14, 21, 22]. As a result, we believe that it is of utmost importance to reflect not only the functional but also the oncological outcome of this patient cohort to get a better understanding of the risk–benefit ratio of future patients.

Thereby, we have evaluated the oncological outcome in 239 patients with hypopharyngeal and laryngeal cancers to evaluate potential differences and secondarily to create a nomogram based on those risk factors to better predict oncological outcome. Hypopharyngeal carcinomas are considered to have the worst prognosis among head and neck cancers with a 5-year OS of around 30–50% compared to 40–60% in advanced stage laryngeal carcinomas with minimal improvement in outcomes among the past two decades [7, 23–25]. A high propensity of lymphatic and systematic spread, predisposition for second head and neck malignancies due to high rates of smoking/alcohol abuse, submucosal spread, high rates of multi-centricity and usual presentation at late tumor stages are assumed as causative factors [26].

This was also reflected by our own cohort demonstrating the poor outcome in hypopharyngeal carcinomas with nodal involvement and salvage situations. Of note, locoregional control and emergence of second cancers were also significantly worse in hypopharyngeal cancers. Submucosal spread and multi-centricity might represent an explanation for the poor locoregional control. The latter has been already linked to combined consumption of alcohol and tobacco use carrying a multiplicative impact, which turned out to be true for our cohort as well [27]. In females, even a moderate consumption of alcohol remarkably increases the risk for hypopharyngeal cancer [28].

We further noticed a significantly higher risk of lymph node involvement in hypopharyngeal tumors. N3 necks occurred in one-third of hypopharyngeal T2 tumors, while N3 neck metastases were almost absent in comparable laryngeal cancer cases. On multivariate analysis, positive neck nodes and salvage procedures represented independent worse prognosticators for outcome. Thereby, anatomic subsite (larynx vs. hypopharynx) poses a significant factor for oncological outcome in univariate but not in multivariate analysis. Consequently, the more aggressive, invasive phenotype of hypopharyngeal tumors, characterized by submucosal spread and a higher rate of lymph node involvement, seems to be associated with the anatomic origin rather than the anatomic subsite itself.

Our data may help to identify patients at higher risk for worse outcome who could benefit from more intensive therapeutic regimes or shorter follow-up intervals. As illustrated by our nomograms, lymph node involvement represented the strongest prognosticator followed by salvage situation and anatomic subsite. We believe that our easily applicable nomogram could be of benefit for future patients and treating physicians as well, for more accurate prediction of outcome. However, we are also aware of the fact that our analysis and pilot nomograms will need to be validated by a second independent test cohort to prove its value. Due to devastating outcome with a 5-year OS of less than 10% in hypopharyngeal patients with positive neck nodes in salvage situation, these patients require the maximum of available treatment options.

The creation of the pilot nomograms as well as the large patient cohort represent strengths of our study; however, there are some limitations of our data as well. First, sociodemographic data (age, sex, BMI) and N-classification did significantly differ between hypopharyngeal and laryngeal cancers. Although this has been described in other studies it may limit drawn conclusions. Second, the retrospective study design always carries an inherent risk of information bias. Finally, the indication for ablative surgeries have changed within the past three decades related to diverse landmark papers showing similar outcome after primary chemoradiotherapy [4–6]. Consequently, the overall number of surgeries have decreased with the number of salvage procedures increased over time, which represents a selection bias.

## Conclusions

Hypopharyngeal cancers are characterized by a more aggressive oncological behavior with worse locoregional control, higher rates of lymph node involvement and poor outcome, which causes a worse outcome compared to laryngeal tumors. This is also depicted by our nomogram which may not only help clinicians to decide if patients may benefit from more aggressive treatment regimens but may also help to better inform patients regarding expectable outcome. However, further studies are necessary to evaluate the reliability of our newly proposed nomogram in larger patient cohorts.

## Data Availability

The data sets used and/or analyzed during the current study are available from the corresponding author on reasonable request.

## Declarations

### Meeting information

No preliminary results have been presented so far.
